# Untangling the genetic link between type 1 and type 2 diabetes using functional genomics

**DOI:** 10.1038/s41598-021-93346-x

**Published:** 2021-07-06

**Authors:** Denis M. Nyaga, Mark H. Vickers, Craig Jefferies, Tayaza Fadason, Justin M. O’Sullivan

**Affiliations:** 1 Liggins Institute, The University of Auckland, Auckland, New Zealand; 2 Starship Children’s Health, Auckland, New Zealand; 3 The Maurice Wilkins Centre, The University of Auckland, Auckland, New Zealand

**Keywords:** Gene regulatory networks, Gene regulation, Chromatin structure, Genetic variation, Transcriptional regulatory elements

## Abstract

There is evidence pointing towards shared etiological features between type 1 diabetes (T1D) and type 2 diabetes (T2D) despite both phenotypes being considered genetically distinct. However, the existence of shared genetic features for T1D and T2D remains complex and poorly defined. To better understand the link between T1D and T2D, we employed an integrated functional genomics approach involving extensive chromatin interaction data (Hi-C) and expression quantitative trait loci (eQTL) data to characterize the tissue-specific impacts of single nucleotide polymorphisms associated with T1D and T2D. We identified 195 pleiotropic genes that are modulated by tissue-specific spatial eQTLs associated with both T1D and T2D. The pleiotropic genes are enriched in inflammatory and metabolic pathways that include mitogen-activated protein kinase activity, pertussis toxin signaling, and the Parkinson’s disease pathway. We identified 8 regulatory elements within the *TCF7L2* locus that modulate transcript levels of genes involved in immune regulation as well as genes important in the etiology of T2D. Despite the observed gene and pathway overlaps, there was no significant genetic correlation between variant effects on T1D and T2D risk using European ancestral summary data. Collectively, our findings support the hypothesis that T1D and T2D specific genetic variants act through genetic regulatory mechanisms to alter the regulation of common genes, and genes that co-locate in biological pathways, to mediate pleiotropic effects on disease development. Crucially, a high risk genetic profile for T1D alters biological pathways that increase the risk of developing both T1D and T2D. The same is not true for genetic profiles that increase the risk of developing T2D. The conversion of information on genetic susceptibility to the protein pathways that are altered provides an important resource for repurposing or designing novel therapies for the management of diabetes.

## Introduction

Type 1 diabetes (T1D) and type 2 diabetes (T2D) are both complex polygenic metabolic disorders, which are generally considered to be pathophysiologically and genetically distinct entities. However, there is some evidence pointing towards T1D and T2D sharing common etiological features (*e*.*g*. apoptosis of pancreatic islet beta cells) resulting in insulin deficiency^1–3^. In young adults, the increase in obesity rates is making it difficult to differentiate between T1D and T2D^4^. Moreover, the latent autoimmune diabetes in adults (LADA) phenotype appears to be an intermediate phenotype that refers to individuals who initially have clinical features that are similar to T2D, but develop autoimmunity towards islet cells leading to progressive beta-cell failure late in life^5,6^. Collectively, these observations may support an overlap in the pathogenesis of both T1D and T2D, but whether this is due to environmental, genetic, or biological pathway intersections, or a combination of these effects remains to be determined.

Comprehensive genome-wide association studies (GWAS) have uncovered distinct and shared loci, marked by single nucleotide polymorphisms (SNPs), which are associated with the development of T1D and T2D^7–10^. Interestingly, Li et al.^11^ suggested that this possible genetic interplay between T1D and T2D could be mediated by the human leukocyte antigen (HLA) region. The HLA locus, which accounts for ~ 50% of the genetic risk for T1D^12^, has been associated with both T2D susceptibility^13,14^ and T2D protection^15^. Furthermore, genetic variants within the transcription factor 7-like 2 (*TCF7L2*) have been strongly associated with T2D and LADA^16,17^, yet the clinical presentation of LADA is similar to T1D (i.e. autoantibody positivity in LADA patients)^17,18^.

The hypothesized existence of shared genetic features in individuals with T1D and T2D indicates that loci act to predispose or protect individuals to one or both of the phenotypes of diabetes—either cumulatively or inversely^19,20^. Such a scenario is not unexpected if one considers the regulation of the insulin secretion, signaling and response pathways as a whole and not as separate modules. Thus, even though the risk alleles for both T1D and T2D may be different, the impacts on gene regulation and biological pathways may converge in both phenotypes: as ultimately diabetes is caused by a lack of insulin action (a relative or absolute deficiency^21^).

We have previously reported that SNPs associated with T2D mark regulatory loci that physically interact with—and act as expression quantitative trait loci (eQTLs) for—genes involved in the leptin and insulin signaling pathways^22^. Furthermore, we subsequently demonstrated that SNPs associated with T1D spatially regulate the expression of genes involved in immune system activation and responses^23^. As such, a greater understanding of how differences in gene regulation contribute to the observed etiological and pathophysiological similarities between T1D and T2D would aid in the management and treatment of diabetes. Here, we characterize the biological pathway overlaps for genes regulated by unique and shared SNPs associated with genetic risk for the development of T1D and T2D.

## Results

### T1D and T2D-associated SNPs form an overlapping gene regulatory network

Previously, we reported that SNPs associated with the development of T1D and T2D mark gene regulatory elements that modulate gene transcript levels^22,23^. Here, we sought to investigate if T1D and T2D share tissue-specific regulatory networks. Emerging evidence indicates that complex diseases culminate from systems-level perturbations^24,25^. Therefore, using the CoDeS3D algorithm^26^ (Methods), we integrated extensive chromatin interaction (Hi-C; Supplementary Table S1) and eQTL data across multiple human tissues. We identified 1,796 and 2,831 unique pairs of spatial eQTLs (T1D: Supplementary Table S2; T2D: Supplementary Table S3; Supplementary Fig. S1) involving 346 and 1,569 T1D and T2D GWAS SNPs at FDR < 0.05, respectively (Supplementary Table S4; Supplementary Table S5). Consistent with our earlier observations^22,23^, ontological analyses (using the R software package; g:Profiler^27^ [Methods]) of the genes that were impacted by the spatial eQTLs identified significant enrichment in immune system response and metabolic signaling pathways (FDR < 0.05), for T1D and T2D, respectively (Supplementary Table S6; Supplementary Table S7).

**Figure 1:**
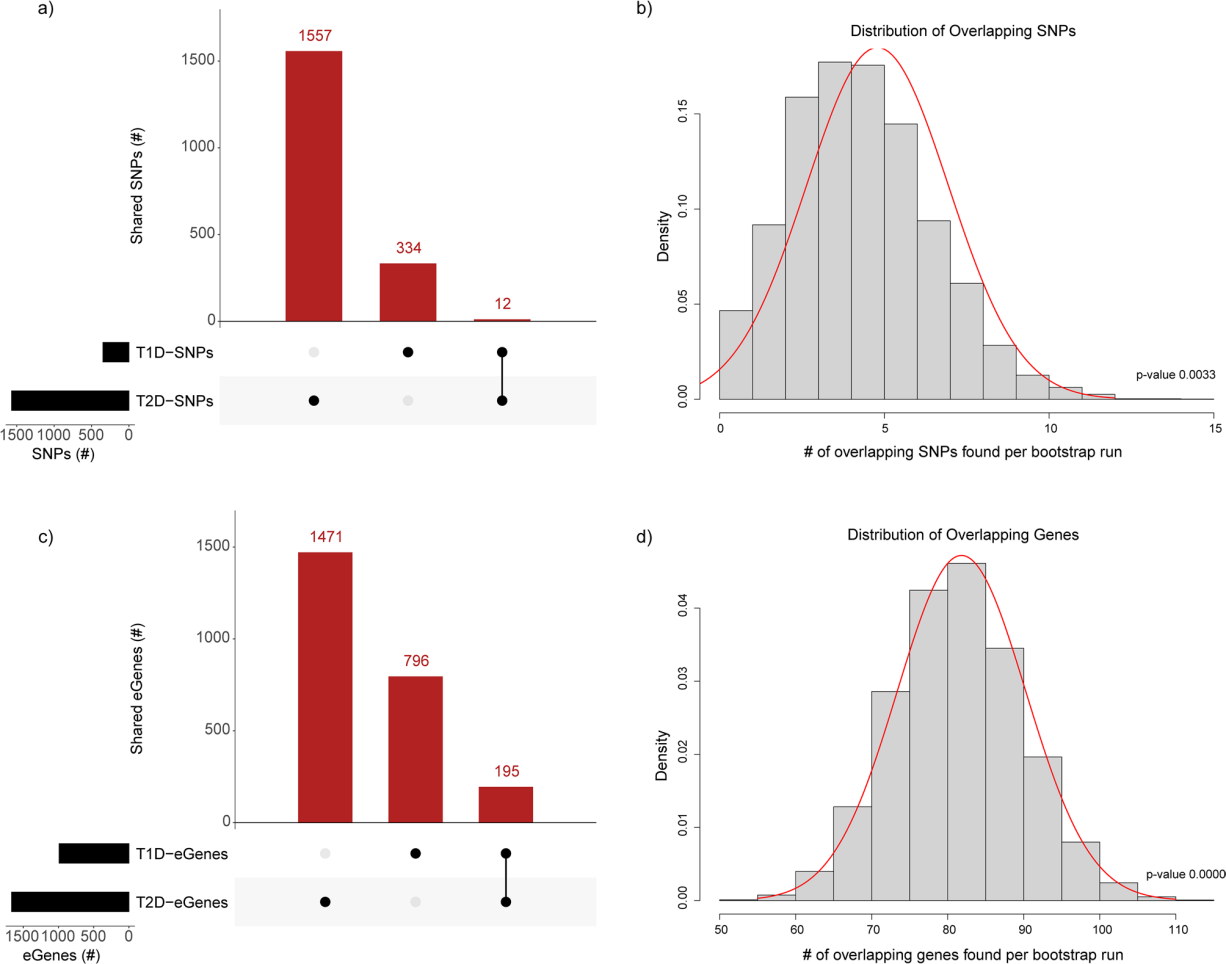
The co-regulation of pleiotropic genes associated with T1D and T2D is non-random. (**a**) A plot showing the number of unique and shared SNPs (imputed and genotyped) associated with the development of T1D and T2D identified from genome-wide association studies. (**b**) A normal distribution plot for randomly sampled SNPs (bootstrapping; n = 10,000) validates the significance of overlaps seen in (**a**). (**c**) The numbers of unique and shared genes modulated by T1D and T2D regulatory SNPs. (**d**) A normal distribution plot for randomly sampled genes (bootstrapping; n = 10,000) validates the significance of overlaps seen in (**c**). eGene—a gene whose transcript levels is associated with an eQTL, eQTL—SNPs associated with transcript levels of genes. Red lines in (**b**) and (**d**) illustrates the fitted normal distribution lines.

It is possible that there is heterogeneity in the groups of SNPs obtained from the GWAS Catalog such that particular SNPs are associated with complications for all forms of diabetes (e.g. LADA and fulminant T1D). The inclusion of SNPs associated with complications in each of the T1D and T2D SNP sets may cause spurious results due to the existence of the identical SNP. Therefore, we tested for the presence of SNPs that were repeated in the T1D and T2D SNP sets we obtained from the GWAS catalogue. We identified 12 identical GWAS SNPs (i.e. 3.5% and 0.8% of T1D and T2D SNPs, respectively) that were present in both datasets (Fig. 1a). Bootstrapping indicated that this overlap does not occur by chance (Fig. 1b), consistent with the idea of shared complications. Notably, 7 of the 12 identical GWAS SNPs were spatial eQTLs (Supplementary Fig. S2).

Colocalization analyses test if two signals (i.e. disease vs cis-eQTL or trait vs trait) share the same ‘causal’ gene or SNP^28^. Bayesian colocalization tests between spatial *cis*-eQTL signals and disease-associated signals were conducted for genomic regions marked by the 7 identical SNPs that were strongly associated (*p* < 10^–8^) with both T1D and T2D (i.e. *SH2B3*, *MAPK14*, *CTRB1/2*, *INS*, *ASCL2/MIR4686*, and *HLA* region). There was no evidence of complete colocalization between the disease and eQTL signals as defined by PP3 + PP4 ≥ 0.99 and PP4/ PP3 ≥ 5, a cut off previously suggested^29^ (Methods; Supplementary Table S8). However, we found weak evidence for colocalization between eQTL and GWAS signals for *CTRB1/2* loci (rs7202877; posterior probability = 35.2%) in both diseases, and *SH2B3* loci (rs3184504; posterior probability = 34.7%) in T1D (Supplementary Table S8). Only 3 regions associated with both traits (i.e. *CTRB1/2*, *SH2B3*, and HLA loci) were found to share a causal SNP (Supplementary Table S8), indicating that T1D and T2D are driven by independent genetic signals. Collectively, this is consistent with the fact that 5 are associated with diabetic foot ulcers, 4 are associated with latent autoimmune diabetes (as well as associated with primary T1D), and 3 associated with primary T1D or T2D).

We reasoned that T1D and T2D would share features that are due to regulatory effects on common pleiotropic genes by SNPs specific to each condition. We identified a total of 195 shared genes (20% and 12% of T1D and T2D genes, respectively) that were modulated by spatial eQTLs associated with T1D and T2D (Fig. 1c; Supplementary Fig. S2). Only 48 shared genes resulted from the 7 eQTLs that were due to identical SNPs. Bootstrapping confirmed that the observed overlap of 195 genes was non-random (Fig. 1d), consistent with the hypothesis that the regulatory effects are on genes that have pleiotropic effects on T1D and T2D.

We examined the 195 shared genes to identify which biological pathways and processes they are involved in. Notably, the subset of 165 shared genes, which excluded 30 classical and non-classical HLA genes, were enriched for pathways that include mitogen-activated protein kinase (MAPK), pertussis and Parkinson’s disease pathways (Table 1). MAPK activity is important in the regulation of pancreatic beta cell function and insulin signaling^30–32^, and beta cell death through inflammatory responses in islet cells^33^. Additionally, pertussis toxin has been implicated in the regulation of insulin secretion from pancreatic beta cells through heterotrimeric G proteins^34–36^. Finally, α-Synuclein, a protein central to Parkinson’s disease^37,38^, has been shown to regulate insulin secretion in beta cells^39^. Collectively, our results support the hypothesis that spatial gene regulatory networks contribute to shared genetic risk between T1D and T2D.

**Table 1:** Significant biological and functional enrichment for pleiotropic genes associated with T1D and T2D.

id	Source	Term id	Term name	Term size	Intersection size	Corrected *p* values
1	CORUM	CORUM:6307	RAB27A-SLP3-KLC1 transport complex	3	2	0.049
2	GO:MF	GO:0005524	ATP binding	1499	24	0.023
3	GO:MF	GO:0035639	Purine ribonucleoside triphosphate binding	1845	27	0.033
4	GO:MF	GO:0032559	Adenyl ribonucleotide binding	1557	24	0.042
5	GO:MF	GO:0030554	Adenyl nucleotide binding	1568	24	0.046
6	GO:MF	GO:0004707	MAP kinase activity	14	3	0.049
7	KEGG	KEGG:05133	Pertussis	76	5	0.016
8	WP	WP:WP2371	Parkinson’s Disease Pathway	38	4	0.032

Corum, comprehensive resource of mammalian protein complexes; MF, molecular function; KEGG, Kyoto Encyclopedia of Genes and Genomes; WP, WikiPathways.

### SNPs used in polygenic risk scores for T1D and T2D modulate transcript levels of genes with pleiotropic effects

**Figure 2:**
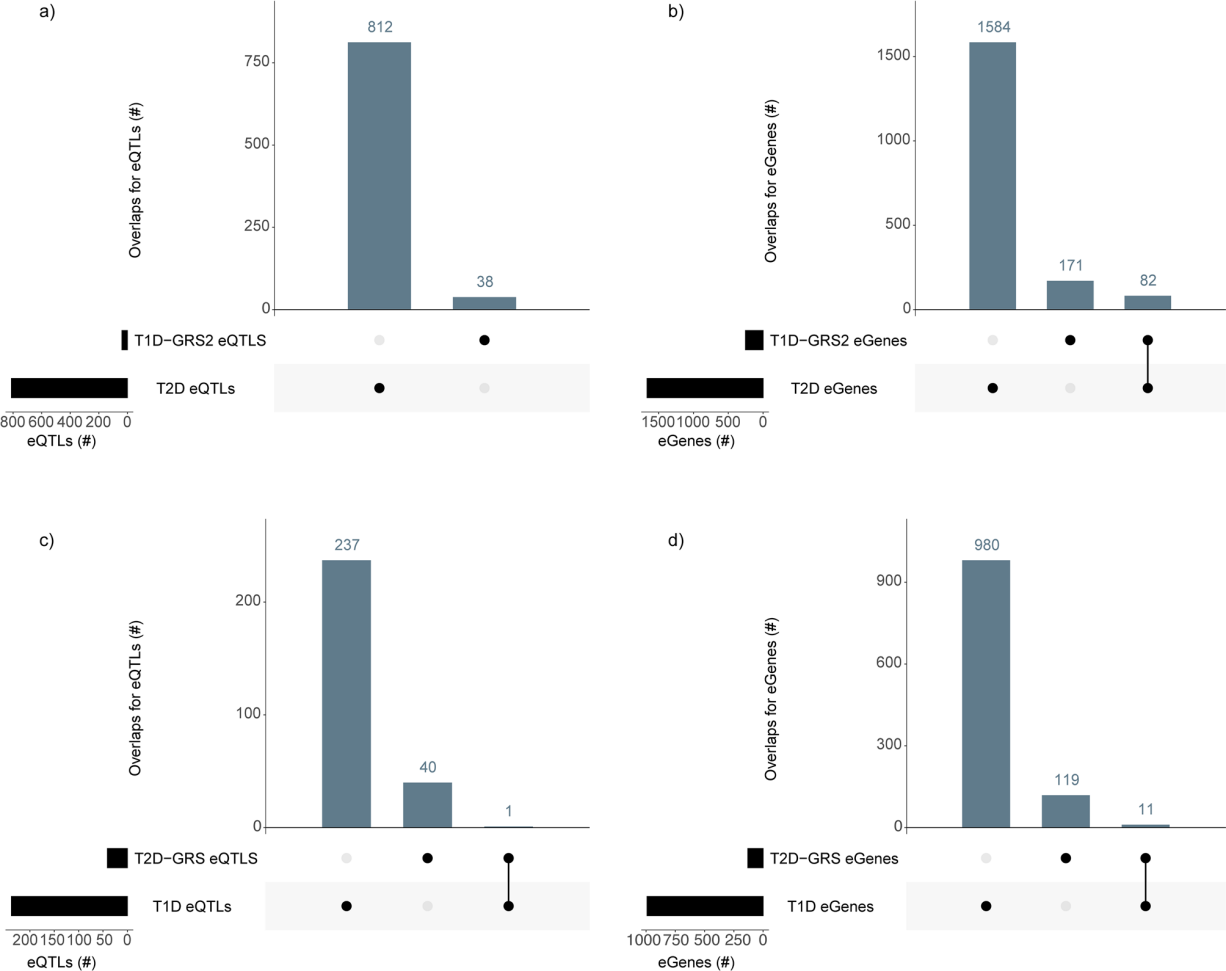
Highly predictive SNPs in polygenic risk scores for T1D and T2D are involved in transcriptional co-regulation of genes that mediate pleiotropic effects. (**a**) The number of unique and shared eQTLs for T1D-GRS2 and T2D SNPs. (**b**) The number of unique and shared genes regulated by spatial regulatory elements marked by T1D-GRS2 and T2D eQTLs. (**c**) The number of unique and shared eQTLs for T2D-GRS and T1D SNPs. (**d**) The number unique and shared genes regulated by regulatory elements marked by T2D-GRS and T1D eQTLs. T1D-GRS2—type 1 diabetes genetic risk score 2, T2D-GRS—type 2 diabetes genetic risk score.

It remains possible that the inclusion of GWAS for, and SNPs associated with, diabetic complications in both the T1D and T2D SNP sets drives the common features we observed within the spatial-eQTLs (Section “T1D and T2D-associated SNPs form an overlapping gene regulatory network”). Therefore, we sought to understand whether highly predictive SNPs used in polygenic risk scores for T1D and T2D^40,41^ are involved in transcriptional co-regulation of genes associated with both diseases. In a polygenic risk score analysis for T1D, Sharp et al*.* included 67 imputed and genotyped T1D SNPs to predict early-onset T1D with 96% accuracy (i.e. T1D genetic risk score 2 [T1D-GRS2])^40^. There were no identical SNPs between the TID-GRS2 and the T2D-associated SNPs lists (Supplementary Table S9). From our CoDeS3D analysis, we found that 38 of the 67 SNPs from T1D-GRS2 are spatial eQTLs that mark regulatory regions for 253 genes (Fig. 2a, b; Supplementary Table S10). Notably, despite no overlap between the T1D-GRS2 and T2D spatial eQTLs (Fig. 2a), we identified 82 shared genes (excluding HLA genes) that were associated with both T1D and T2D (Fig. 2b).

In a polygenic risk score analysis for T2D (T2D-GRS), 62 T2D-associated SNPs were combined with age, sex, and clinical risk factors to predict T2D development with 91% accuracy^41^. Two SNPs were identical between the T2D-GRS and the T1D-associated SNP lists (Supplementary Table S9). From our CoDeS3D analysis, we identified that 41 of the 62 SNPs from the T2D-GRS mark spatial regulatory elements for 130 genes (Fig. 2c,d; Supplementary Table S11). Notably, we identified 11 shared genes between T2D-GRS and the T1D-associated eQTLs (Fig. 2d), of which 9 were associated with the identical eQTL (rs7202877; Fig. 2c).

It is notable that the comparison of genes associated with T1D-GRS2 vs T2D-associated eQTLs showed greater overlap than the comparison of genes associated with T2D-GRS vs T1D-associated eQTLs (Fig. 2b, d, respectively). Collectively, our findings are consistent with the hypothesis that the highly predictive SNPs used in polygenic risk scores for T1D are involved in transcriptional co-regulation of genes that mediate pleiotropic effects in both T1D and T2D.r

### T1D and T2D variant heritability is not significantly correlated in Europeans

We observed that GWAS SNPs associated with T1D and T2D are involved in transcriptional co-regulation of pleiotropic genes from the CoDeS3D analysis. Therefore, we employed the high-definition likelihood (HDL) method^42^ to calculate the genetic correlation between variant effects on T1D and T2D risk using European ancestral summary data from the UK Biobank (UKBB). The HDL method robustly increases the precise estimation of genetic correlation between phenotypes, and estimates variant heritability, through its extensive inclusion of genome-wide linkage disequilibrium^42^. HDL analysis did not identify a genetic correlation between T1D and T2D (*r*_*g*_ = 0.17; *p* value = 5.9 × 10^–2^) (Table 1). T1D did not correlate with either body mass index, or obesity (Table 2). However, consistent with previous observations by Carlsson et al.^43^, we observed a significant positive correlation between T2D and body mass index, and between T2D and obesity (Table 2). These results are consistent with the interpretation that the overlap of biological mechanisms between T1D and T2D occurs at the level of gene control and not at the level of variant heritability.

**Table 2:** Genetic correlation estimates between T1D, T2D, body mass index and obesity in people of European ancestry.

Phenotype 1	Phenotype 2	*r*_*g*_ (s.e.)	*p* value
Type 1 diabetes	Type 2 diabetes	0.17 (0.09)	5.9 × 10^–2^
Type 1 diabetes	BMI	0.04 (0.03)	2.2 × 10^–1^
Type 1 diabetes	Obesity	0.08 (0.09)	4.4 × 10^–1^
Type 2 diabetes	BMI	0.48 (0.04)	5.5 × 10^–40^*
Type 2 diabetes	Obesity	0.31 (0.09)	7.9 × 10^–4^*
Obesity	BMI	0.65 (0.06)	2.9 × 10^–25^*

*r*_*g*_, genetic correlation estimate; s.e., standard error; *p* value, Bonferroni corrected *p* values; *significant corrected *p* values. Variant heritability estimates (h2; s.e.) are: T1D (0.0046; 9e-04), T2D (0.01; 9e-04), BMI (0.2565; 0.0081), Obesity (0.0061; 0.0011).

### The *TCF7L2* locus is a spatial regulatory hub for genes important for immune regulation and T2D etiology

**Figure 3:**
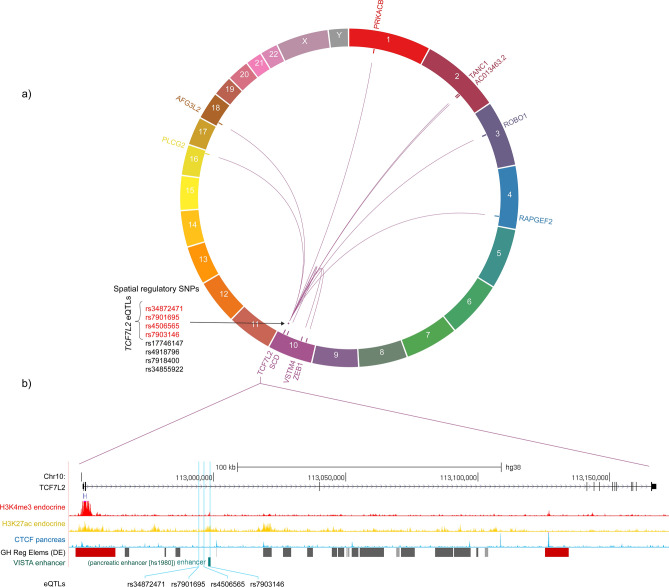
Regulatory SNPs within the *TCF7L2* locus modulate the expression of genes involved in immune regulation and genes important for the etiology of T2D. (**a**) A circos plot of significant regulatory interactions (i.e. innermost link lines) between SNPs within the *TCF7L2* locus (i.e. the pointed purple dot) and the spatially regulated genes (i.e. labelled genes in the outermost track) at FDR < 0.05. Spatial regulatory SNPs modulating the expression of *TCF7L2* are highlighted. Linkage disequilibrium between genetic variants was obtained from https://ldlink.nci.nih.gov/. (**b**) An expanded view of the *TCF7L2* gene locus. SNP rs7903146 overlaps histone modification marks (i.e. pancreatic H3K4me3 and H3K27ac) and an annotated enhancer in the pancreas. Genome regulatory tracks were obtained from UCSC browser using hg38 coordinates chr10:112945186-113172435 (https://genome.ucsc.edu). GH Reg Elems (DE)—GeneHancer regulatory elements (double elite). Circa software was used to generate the circos plot and is available at http://omgenomics.com/circa/. The annotated pancreatic enhancer (hs1980) was extracted from VISTA enhancer database^94^.

*TCF7L2* is a knownT2D susceptibility locus^44^ that encodes a transcription factor that is central to the Wnt signaling pathway. SNPs mapped within *TCF7L2* have also been associated with the presence of islet autoantibodies in LADA^6,16–18^ and recent-onset T1D patients^45^. As such, *TCF7L2* has been hypothesized to be the key to understanding the genetic link between the pathogenesis of T1D and T2D^20^. We hypothesized that *TCF7L2* is a spatial regulatory hub for genes important for the etiology of T1D and T2D. Our CoDeS3D analysis identified 8 regulatory elements marked by SNPs within the *TCF7L2* locus (Supplementary Table S12). Four of these eQTLs (i.e. rs34872471, rs7901695, rs4506565, rs7903146) coordinate the regulation of *TCF7L2* expression (Fig. 3a; Supplementary Table S12). Notably, rs4506565 is associated with single autoantibody in recent-onset T1D^45^. The 4 regulatory SNPs are also in strong linkage (R^2^ > 0.8) across the European population. Rs7903146 overlaps histone modification marks and an annotated enhancer in the pancreas (Fig. 3b).

Notably, our CoDeS3D analysis identified regulatory elements within the *TCF7L2* locus that were associated with *trans*-regulation of genes involved in the regulation of immune responses, including *PLCG2, ZEB1,* and *ROBO1* (Fig. 3; Supplementary Table S12). *PLCG2* encodes a phospholipase implicated in inflammation and autoimmunity^46^, and in T cell function and selection^47^. *ROBO1* expression has been hypothesized to serve as a biomarker for T1D diagnosis due to its regulatory role in the recruitment of diabetogenic T cells^48^. Additionally, *ZEB1*, which is also spatially regulated in *cis* by a T1D-eQTL (i.e. rs2793108—81 Mb away from the *TCF7L2* locus; Supplementary Table S2), encodes a zinc finger transcription factor that functions as a key regulator of the T cell signaling and differentiation in the thymus^49^.

Interestingly, we also identified a SNP within *TCF7L2* (i.e. rs17746147) that modulated the expression of genes involved in insulin signaling (i.e. *SCD*) (Supplementary Table S12). Stearoyl-CoA desaturase is encoded by *SCD* and catalyzes the biosynthesis of monounsaturated fatty acids. Notably, stearoyl-CoA desaturase has been implicated in insulin resistance (IR) together with *TCF7L2*^50,51^. Collectively these results support the hypothesis that the *TCF7L2* locus acts as a regulatory hub for genes involved in immune regulation as well as genes important in the etiology of T2D.

### Cross-tissue eQTL enrichment of associations in T1D and T2D

**Figure 4:**
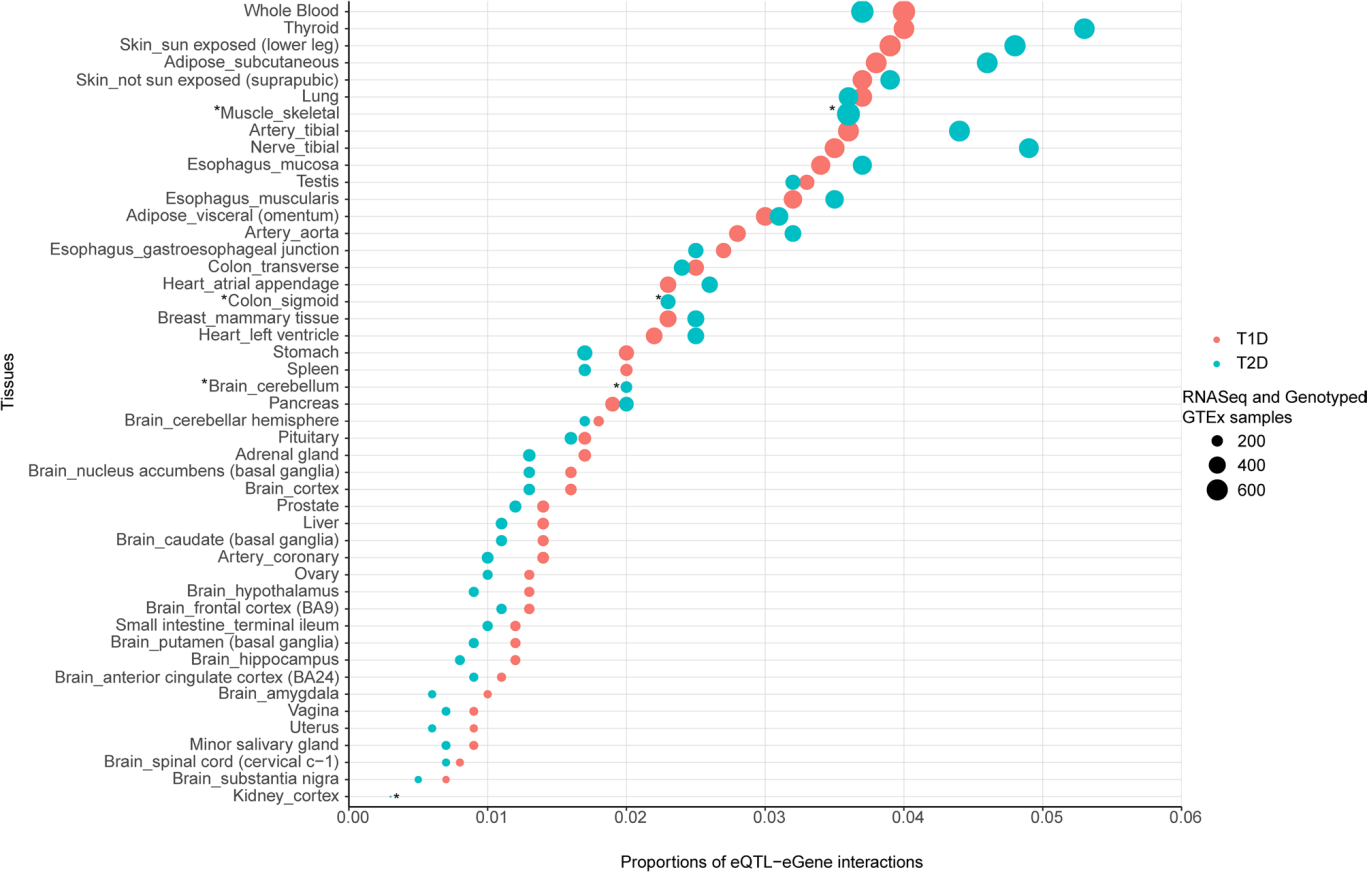
Functional eQTL effects are variably distributed across tissues. The proportions of significant T1D and T2D eQTL-eGene interactions across tissues (x-axis) in comparison to number of GTEx tissue samples (circles). The top ranked tissues with the highest number of functional T1D eQTL-eGene interactions include whole blood, thyroid, skin and adipose tissues. T2D eQTLs were greatest in the thyroid, tibial nerve, skin and adipose subcutaneous tissues. *Tissues with the same number of functional T1D and T2D eQTL-eGenes interactions.

We mapped tissue-specific regulatory networks, leveraging information on eQTL effects from the CoDeS3D analysis (Supplementary Table S2; Supplementary Table S3), to identify the tissues in which the disease-associated loci are most likely functional (i.e. eQTL-eGene-tissue triads). Consistent with previous observations^22,23^, we found that eQTL effects for T1D and T2D were variably distributed across different tissues (Fig. 4). The top-ranked tissues with the highest number of functional eQTL-eGene interactions for T1D SNPs included whole blood, thyroid, skin, and adipose subcutaneous tissues (Fig. 4). Thyroid, tibial nerve, skin and adipose subcutaneous tissues had the greatest numbers of regulatory impacts involving T2D eQTLs (Fig. 4). Tissue-specific enrichment analysis using TissueEnrich (R package) identified thyroid tissue as having the highest level of enrichment for expression of the genes that were regulated by eQTLs associated with T2D, while lymph nodes, lung and spleen were the most enriched tissues for genes regulated by T1D eQTLs (Supplementary Fig. S3).

**Figure 5:**
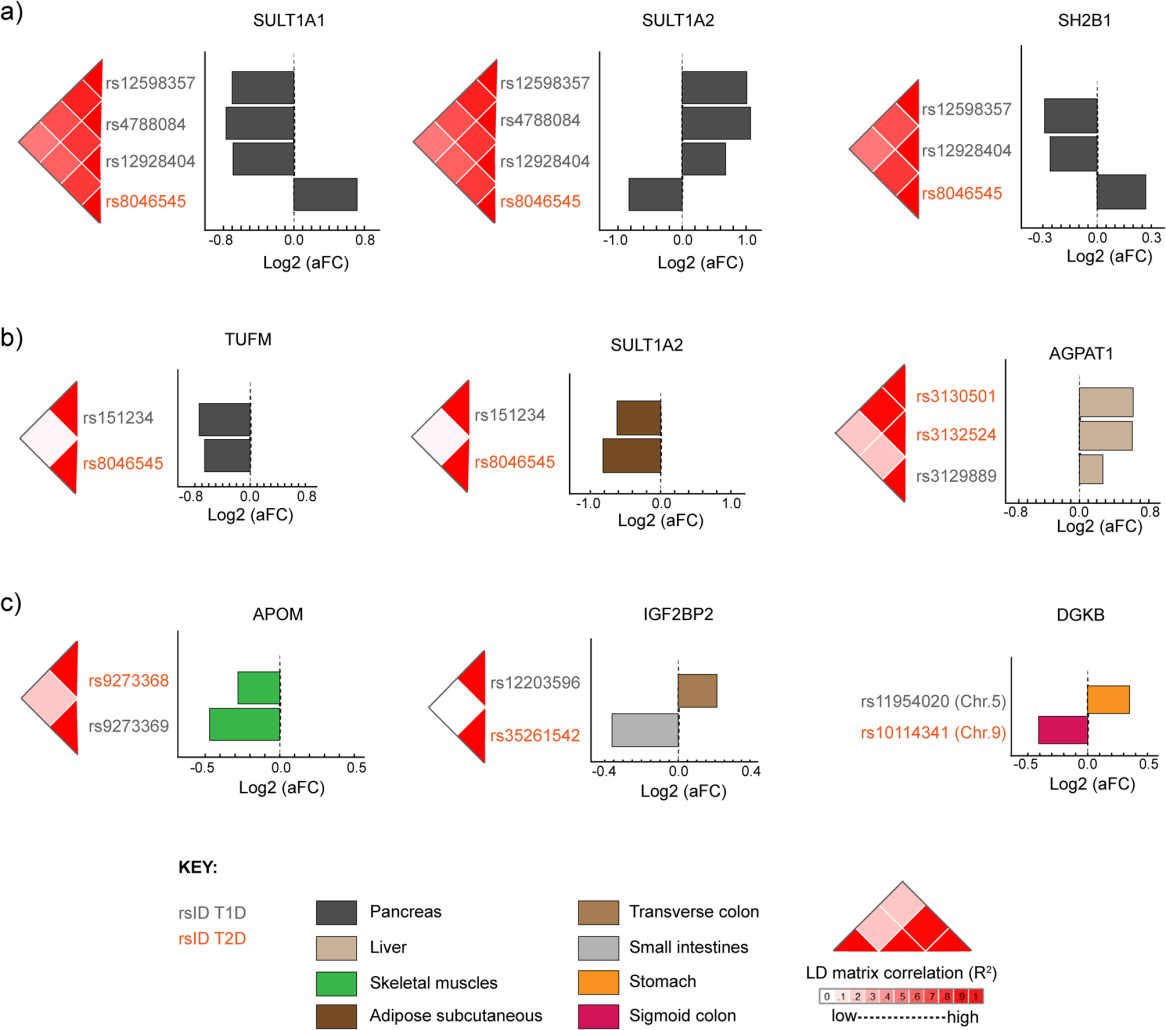
T1D and T2D eQTLs have tissue-dependent effects across human tissues. (**a**) T1D and T2D *cis*-eQTLs are associated with spatial regulation of shared genes in the pancreas but in opposite directions. (**b**) T1D and T2D *cis*-eQTLs have similar eQTL effects on shared genes across tissues. (**c**) Trans-eQTLs have mixed effects on transcript levels of shared genes across tissues. aFC—allelic fold change, which denotes the direction of eQTL effect [i.e. upregulated (+) or downregulated (−)]. cis-interactions within 1 Mb on the same chromosome; trans-interactions > 1 Mb either on the same chromosome or interactions > 1 Mb on different chromosomes. Linkage matrices between SNPs are based on European population and were obtained from https://ldlink.nci.nih.gov/.

It has been recently demonstrated that eQTLs associated with complex traits can have opposing effects on gene regulation in different tissues^52^. Therefore, we sought to determine whether T1D and T2D eQTL effects on the 165 pleiotropic genes (excluding 30 HLA genes) occurred in the same or opposite directions. We observed that a number of eQTLs impacted on the expression of shared genes in opposing directions across the same tissues (Supplementary Table S13). For example, T1D *cis*-eQTLs rs12598357, rs12928404, and rs4788084 downregulated *SULT1A1* transcript levels in the pancreas. By contrast, a T2D *cis*-eQTL rs8046545 upregulated *SULT1A1* in the pancreas (Fig. 5a). Similarly, *SULT1A2* pancreatic transcript levels were upregulated by T1D *cis*-eQTLs (i.e. rs12598357, rs12928404, rs4788084). Again, the T2D-associated *cis*-eQTL (i.e. rs8046545) downregulates *SULT1A2* pancreatic transcript levels (Fig. 5a). Of the *SULT1A2* transcript levels regulating eQTLs, only rs8046545 and rs12928404 are in strong LD (R^2^ > 0.78) in people of European ancestry (Fig. 5a).

We also identified instances where T1D and T2D eQTLs modulate the transcript levels of shared genes in the same direction. For example, both rs151234 (T1D eQTL) and rs8046545 (T2D eQTL) downregulated the expression of *SULT1A2* and *TUFM* in adipose and pancreas, respectively (Fig. 5b). In addition, both rs3130501 and rs3132524 (T2D eQTLs), together with rs3129889 (T1D eQTL), co-modulate the expression of *AGPAT1* in the same direction in the liver (Fig. 5b). Notably, rs151234 is not in linkage with rs8046545 (R^2^ < 0.1), consistent with the SNPs marking distinct spatial regulatory elements that are not co-inherited.

We observed that *trans*-eQTLs have mixed effects on transcript levels of shared genes. For example, rs12203596 (T1D eQTL) upregulated *IGF2BP2* in the transverse colon, while rs35261542 (T2D eQTL) downregulated *IGF2BP2* expression in the terminal ileum tissue (Fig. 5c). By contrast, *APOM* expression was downregulated by rs9273368 (*trans*-eQTL associated with T1D, T2D, and LADA) and rs9273369 (*trans*-eQTL associated only with T1D) in skeletal muscle tissues (Fig. 5c). Notably, rs9273368 and rs9273369 are strongly co-inherited (R^2^ > 0.8) in the African Yoruba population but not people of other ancestries.

Collectively, these results indicate that: (a) eQTL effects for T1D and T2D SNPs have tissue-specific effects on gene expression; and (b) T1D and T2D SNPs can co-regulate genes in the same tissue consistent with the existence of converging biological pathways.

### Protein–protein interaction network identifies drug repurposing targets

**Figure 6:**
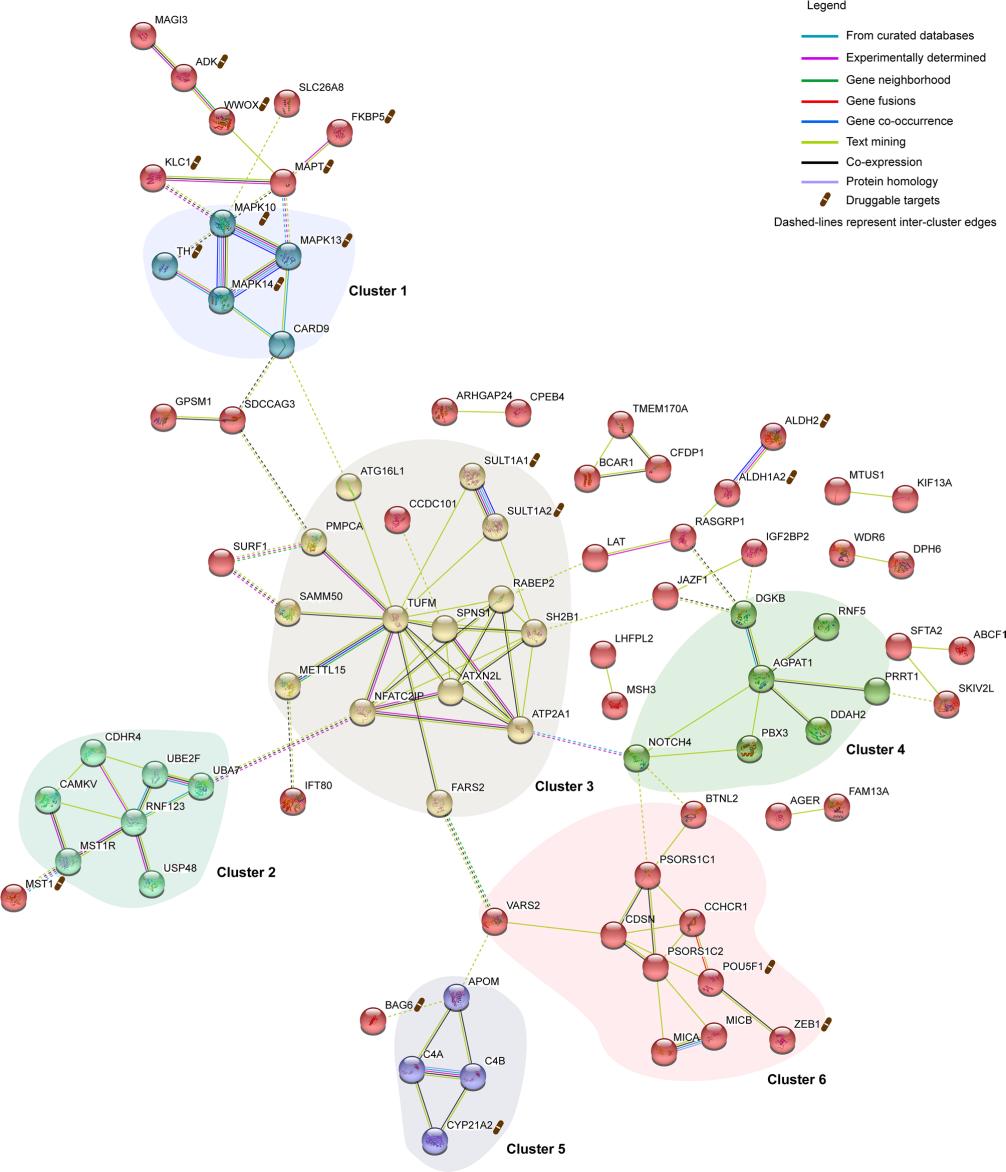
Protein–protein interaction network identifies existing drug targets that may impact diabetes. A protein–protein interaction network for the pleiotropic genes was constructed using string-db-org (http://string-db.org; version 11^53^) set to a medium confidence of 0.400 and prediction methods (i.e. genomic context prediction channels—neighbourhood, fusion, and co-occurrence; together with co-expression, text mining, curated databases, and experiments). 137 nodes (i.e. genes encoding functional proteins) were identified from an input of 165 genes at a PPI enrichment of *p* < 1.0 × 10^–16^. Nodes with at least 2 interactions are shown. PPI clusters were identified using K-means clustering algorithm set to 6 clusters (K-means = 6). Dashed lines represent inter-cluster edges. Proteins with a drug symbols are targets for existing FDA-approved drugs from the Drug Gene Interaction database (DGIdb; v3.0.2). A summary of the protein interaction network with the respective interaction scores is available in Supplementary Table S14. A summary of the drug-gene interactions from DGIdb is available in Supplementary Table S16.

Traits that share core genes or whose genes interact closely in biological pathways are hypothesized to have correlated effects^25^. Therefore, we used STRING (Methods; http://string-db.org; version 11^53^) to construct the protein–protein interaction (PPI) network for the 165 shared genes associated with both T1D and T2D. Of the 165 genes analyzed, we identified 137 nodes (i.e. functional proteins encoded by the genes) and 117 edges (i.e. predicted functional associations) at a significant PPI enrichment of *p* < 1.0 × 10^–16^ (Fig. 6; Supplementary Table S14). STRING identified 6 PPI clusters (circled) using the K-means clustering algorithm (K-Means = 6) of functional biological interactions within the overall network (Methods; Fig. 6; Supplementary Table S15). These clusters included, for example, the hub of highly connected genes (i.e. *METTL15, SAMM50, PMPCA, SH2B1* and *ATG16L1*; Fig. 6; Supplementary Table S14) about the *TUFM* gene, which encodes the mitochondrial translation elongation factor. This *TUFM*-associated hub is enriched in regulatory proteins important for mitochondrial function^54–57^, consistent with the central role that mitochondrial dysfunction is hypothesised to have in diabetes^58,59^.

We hypothesized that the proteins within our PPI network represented high value targets for therapeutic development. Therefore, we mined the Drug Gene Interaction database (DGIdb) to identify shared genes that encode proteins that are affected by at least one drug within the interaction networks. We identified that 25 of the 165 genes (~ 15%) encode proteins affected by FDA approved drugs (Supplementary Table S16). The proteins encoded by these genes interact directly with other proteins within the PPI network (Fig. 6). Some of the drugs we identified (e.g. streptozotocin, pembrolizumab, nivolumab, and doxorubicin) cause diabetes or diabetes-like symptoms (i.e. hyperglycemia) as side-effects of usage. For example, streptozotocin affects the proteins encoded by *TH* and *SULT1A2* (Supplementary Table S16), and has been widely used experimentally to induce diabetes in rodent models due to its toxic effects on pancreatic beta cells^60^. Pembrolizumab and nivolumab (targeting the protein encoded by *MSH3*) have been reported to induce the development of insulin-dependent diabetes in cancer patients^61^. Similarly, doxorubicin (targeting the protein encoded by *ZEB1*) has been shown to result in severe hyperglycemia and IR in an experimental rat model^62^ (Supplementary Table S16).

Not all of the side-effects are negative. For example, sirolimus, affects the *MAPK10* gene product, has been shown to normalize glucose metabolism in diabetic mice^63,64^, decrease IR in diabetic rats^65^, and prevent IR in humans^66^ (Supplementary Table S16). Similarly, mitomycin (another compound that affects the *MAPK10* gene product) has been hypothesized to suppress pro-inflammatory events and cause the induction of regulatory T cells differentiation following islet allograft transplantation^67^.

Several of the drugs we identified have been trialed or repurposed for the treatment of diabetes. For example, disulfiram (targeting *ALDH2* gene product) has recently been shown to normalize body weight and restore insulin responsiveness in obese mice^68^. Similarly, pirfenidone and tretinoin (Supplementary Table S16) have been trialed for the management of long-term diabetic complications, i.e. diabetic nephropathy and diabetic foot-ulcers, respectively^69,70^. Collectively, our results highlight the potential for a genetics-informed and network-based approach to understand and prevent adverse reactions while providing an avenue for repurposing existing drugs in the management of diabetes.

## Discussion

We have identified how genetic variation associated with T1D and T2D impacts on common biological pathways through putative gene regulatory networks that include both shared and unique genes. Our data show that spatial eQTLs nearby T1D and T2D associated genetic variants share downstream pathways. Notably, transcript levels of genes associated with eQTLs for the highly discriminatory PRS for T1D (T1D-GRS2) showed greater overlap with T2D-associated eQTLs than was observed when comparing transcript levels of genes associated with T2D-GRS and T1D-associated eQTLs. Yet, there was no significant genetic correlation detected in people of European ancestry (using UKBB data). Thus, our findings are consistent with forms of T1D and T2D having overlapping biological mechanisms that arise from regulatory impacts on shared genes and pathways. However, it appears that a genetic risk profile for T1D alters more biological pathways that increase the risk of developing both T1D and T2D, than the reverse.

It has been suggested that the development of complex ‘related’ traits can be driven by tissue-and disease-specific eQTL effects on the regulation of common genes^52^. Consistent with this, we observed upregulation of *SH2B1* in adipose and pancreatic tissues by a T2D eQTL, and downregulation of *SH2B1* expression by a T1D eQTL across the same tissues. While the eQTLs we identified are associated with a life-long reduction or increase in expression relative to the reference genotype, this is still environmentally modifiable by epigenetic mechanisms^52^. However, it is worth noting that hypothalamic overexpression of *SH2B1* was recently reported in a mouse model to protect against obesity and metabolic disease, including diet-induced IR^71^.

Further support for the impact of disease-specific genetic variation on shared genes is obtained from the opposing T1D and T2D *trans*-eQTL effects on diacylglycerol kinase beta (encoded by *DGKB*), whose kinase family has been implicated in peripheral IR and abnormal glucose uptake^72^. Similarly, T1D- and T2D-specific eQTL effects were observed on *SULT1A1* and *SULT1A2*, which encode enzymes involved in amine metabolism and lipid metabolic pathways^73^. Therefore, we contend that our results support the hypothesis that genetic risk impact tissue-specific regulation of shared genes, thereby influencing the etiology of T1D and T2D through similar metabolic pathways but different mechanisms.

The protein–protein interaction network we identified revealed an intricate metabolic network for the shared genes regulated by both T1D and T2D eQTLs. For example, a hub protein, apolipoprotein M (encoded by *APOM*), is a key regulator of high-density lipoprotein metabolism that subsequently modulates the efflux of cholesterol and atherosclerosis susceptibility^74^. Another hub protein, AGPAT1, together with AGPAT2, has important roles in the biosynthesis of glycerophospholipids and is hypothesized to play a role in the development of IR^75^. Notably, IR is a prominent feature for both T1D and T2D and has been demonstrated to impact on lipid and lipoprotein metabolism, ultimately resulting in dyslipidemia and diabetes-associated vascular complications^76–78^.

Diabetes is a very heterogeneous disease in regard to the clinical, genetic, immunologic, and metabolic features that define disease onset and progression. Notably, genetic risk scores for T1D and T2D have been instrumental in predicting disease-onset^40,41^. However, transcriptional risk scores (TRS) have been reported to outperform genetic risk scores in distinguishing patients with Crohn disease from healthy subjects and predicting disease progression^79^. Therefore, since most disease-associated SNPs regulate transcript levels of genes (which is in a sense closer to the phenotype), understanding how these SNPs influence gene expression is important to identify genes whose association with disease is either through protection, promotion, or pleiotropy^79^. For instance, the autoimmune LADA phenotype is considered a genetic admixture of T1D and T2D due to its association with *TCF7L2*, a transcription factor that is also associated with T2D risk^16^.

Interestingly, our analysis identified spatial regulatory elements within the *TCF7L2* locus associated with the expression of immune regulatory genes, as well as genes involved in insulin signaling pathways. One key finding was the identification that T2D eQTLs, within *TCF7L2*, and a T1D eQTL *trans*-regulate *ZEB1* gene. *ZEB1* encodes a zinc finger transcription factor that functions as a key regulator of the T cell signaling and differentiation in the thymus^49^. Therefore, we contend that the *TCF7L2* locus encompasses a regulatory hub for genes important for the etiology of T1D and T2D. Our conclusion corroborates observations of *TCF7L2* associated gene regulatory impacts^80^ and studies reporting that *TCF7L2* SNPs are associated with the presence of islet autoantibodies in LADA^17^, and autoantibody positivity in recent-onset T1D patients^45^.

Our study identified targets for drugs associated with adverse reactions through the integration of PPI networks and drug-gene interactions. For example, streptozotocin, which targets pleiotropic proteins, has been demonstrated to induce diabetes in rodent models^60^. By contrast, sirolimus is reported to prevent IR in humans^66^. At the same time, the efficacy of pirfenidone and tretinoin has been evaluated in the management of diabetic nephropathy and foot-ulcers, respectively^69,70^. Moreover, disulfiram, which is used for the treatment of alcoholism, has been shown to normalize fat mass and insulin sensitivity in diet-induced obese mice and repurposing of this drug in the clinic has been suggested as a strategy to treat obesity and related metabolic complications^68^. Notably, studies on monogenic forms of diabetes such as neonatal diabetes have provided a proof-of-concept that an individual’s genotype can guide on the treatment modality^81^. Therefore, it seems plausible that genetics-informed and network-based prescription could provide an avenue for repurposing existing drugs while preventing adverse drug reactions.

Our study has limitations. Firstly, our genetic correlation and colocalization analyses were performed using genome-wide genotype data of individuals of the European ancestry, reflecting that over 90% of GWA studies on T1D have been performed in populations of European ancestry. Secondly, the colocalization test assumes a single causal variant for a trait^28^. Moreover, it ignores the fact that transcript levels of genes can be modified through various mechanisms, not all of which are necessarily associated with disease risk^28^. Furthermore, the lack of complete colocalization between disease and eQTL signals could indicate that the right SNP-gene-tissue triads were eliminated from the tests by selecting only the SNP-gene pairs with the lowest *p* values. Nonetheless, our analyses revealed partial colocalization between disease and spatial eQTL signals. Therefore, we contend that experimental manipulation through CRISPR will be required to establish causality. Thirdly, the genetic admixture of GWAS SNPs, together with the inclusion of GWAS SNPs associated with phenotypes that are not classically defined as T1D could limit the generalization of pleiotropic effects. Nonetheless, the inclusion of highly predictive SNPs used in polygenic risk scores for T1D and T2D from populations with fairly similar genetic linkage strongly supports the identified co-transcriptional regulation of shared genes for T1D and T2D. Fourthly, our analysis involved the use of datasets from ‘whole’ pancreatic tissue, which contains a mixture of endocrine and exocrine cells. Future studies should limit their analyses to single cell types to confirm the pleiotropy we identified. Finally, the integration of extensive Hi-C datasets increases the power to detect more cell type and developmental stage-specific functional chromatin interactions to understand the genetic basis of complex diseases at the systems-level. However, as the number of tests increases, correcting for multiple testing using the Benjamini–Hochberg (BH) procedure is conservative^82^. This could potentially result in an under-estimate of the extent of shared gene overlap between T1D and T2D, thereby underestimating the identification of pleiotropic genes. Nevertheless, the BH procedure corrects for multiple testing by ranking *p* values^82^, which ensures a very high probability of true-positives, thereby increasing the confidence of eQTL associations.

## Conclusion

Our findings support the existence of common genetic regulatory mechanisms that co-regulate genes that mediate pleiotropic effects on T1D and T2D. Importantly, our results further support the role of *TCF7L2* locus, a well-known T2D susceptibility region, as a key regulatory hub that modulates transcript levels of genes involved in immune regulation as well as genes important in the etiology of T2D. Empirical studies that integrate genome editing techniques (i.e. CRISPR-Cas9) will further refine our understanding of these regulatory interactions and their roles in the development of islet autoimmunity, T1D and T2D.

## Methods

### Identification of SNPs associated with the development of T1D and T2D

The genetic variants used in this study were genotyped and imputed SNPs associated with T1D obtained from: the GWAS catalog (a keyword search for “Type 1 diabetes” was performed and associations were selected based on a *p* value threshold [*p* values < 5 × 10^–6^]; http://www.ebi.ac.uk/gwas; v1.0.1; downloaded March 25, 2020) (Supplementary Table S4); studies on polygenic risk scores for T1D^40,83,84^; prospective studies^10,85–87^; and time-to-event studies^88,89^. For the T2D-associated genetic variants, SNPs were obtained from the GWAS catalog (a keyword search for “Type 2 diabetes” was performed and associations were selected based on a *p* value threshold [*p* values < 5 × 10^–6^]; http://www.ebi.ac.uk/gwas; v1.0.1; downloaded April 8, 2020) (Supplementary Table S4), and a study on T2D polygenic risk scores^41^. A total of 346 T1D SNPs and 1,569 T2D SNPs were used in the eQTL analysis (Supplementary Table S5). Genomic positions for SNPs are annotated according to reference human hg38 genome build.

### Identification of spatial eQTL-eGene pairs for T1D and T2D-associated SNPs

We used the Contextualizing Developmental SNPs in 3-Dimensions (CoDeS3D) algorithm as described in^26^ to identify SNPs associated with the spatial regulation of gene transcript levels through physical interactions. Briefly, the CoDeS3D modular python scripts integrate Hi-C contact libraries from published sources (Supplementary Table S1) to identify spatial co-localization of two DNA fragments, with one fragment marking the queried SNP. Gene-containing restricted fragments that are in physical contact with fragments containing the queried SNPs are identified as spatial pairs to the SNPs. Finally, the resultant spatial SNP-gene pairs are queried in the Genotype-Tissue Expression database (GTEx) to identify SNPs that are associated with transcript levels of genes through physical interaction at FDR < 0.05^26^.

Here, we integrated extensive Hi-C contact libraries to identify all possible tissue, cell type and developmental stage-specific chromatin interactions based on the emerging evidence that complex diseases culminate from systems-level perturbations^24,25^. First, the spatial interactions were identified from Hi-C contact libraries captured from: (1) primary human tissues (i.e. including pancreas, liver, lung, spleen, muscle, and adrenal gland); (2) primary and immortalized immune cell-types (i.e. B and T lymphocytes); and (3) embryonic stem cells, including cell lines representing embryonic germ layers (Supplementary Table S1). Next, the regulatory potential of the identified SNP-gene pairs was tested through the integration of expression QTL information from 47 human tissues and 2 immortalized cell-lines (Genotype-Tissue Expression database [GTEx] v8; http://www.gtexportal.org^90^).

Spatial eQTLs were deemed significant and recorded if the FDR < 0.05 after correcting for multiple testing using the BH procedure^82^. Finally, genes whose transcript levels were associated with a spatial-eQTL were denoted as eGenes. The eQTL-eGene interactions were defined as either *cis* (i.e. interactions within 1 Mb on the same chromosome), *trans*-intrachromosomal (i.e. interactions > 1 Mb but on the same chromosome), or *trans*-interchromosomal (i.e. interactions > 1 Mb but on the different chromosomes). All datasets and analyses were prepared and carried out using the human genome reference build GRCh38.p7. Genomic positions for eGenes derived from GTEx are annotated according to GENCODE v25. The HLA genes were excluded from the shared genes analyses because we wanted to identify HLA independent key pathways and networks since HLA genes are strongly associated with T1D^12^.

### Genetic correlation and SNP heritability analyses

We employed the recently developed HDL method^42^ to estimate the genetic correlation between T1D and T2D, together with obesity and body mass index (BMI) using population-level data from the UKBB. The genome-wide genotype data available in the UKBB is obtained from a large prospective cohort study of ~ 500,000 individuals across the United Kingdom, providing a rich resource for genetic analyses. Genetic correlation and SNP heritability analyses for the phenotypes in this report were conducted as described on https://github.com/zhenin/HDL/wiki. Briefly, the UKBB summary statistics of genome-wide associations for T1D, T2D, BMI and obesity were obtained from the Neale lab (i.e. round 2 association tests released in 2018; https://www.nealelab.is/uk-biobank/). The association tests on curated phenotypes were performed on 361,194 unrelated individuals of British ancestry as described on https://www.nealelab.is/uk-biobank/. Computed linkage disequilibrium matrices and imputed reference panels of HapMap3 SNPs (i.e. 1,029,876 quality-controlled UKBB imputed SNPs) were downloaded from https://github.com/zhenin/HDL/wiki/Reference-panels. The imputed panel of SNPs was used as it provides a more accurate estimate of genetic correlations ^42^.

### Genetic colocalization analyses

Genetic colocalization analysis permits the identification of shared ‘causal’ SNPs or genes within a genomic loci across disease vs trait or trait vs trait association signals^28^. Bayesian colocalization tests between spatial *cis*-eQTL and disease-associated signals were performed for 7 genomic regions strongly associated (*p* < 10^–8^) with both T1D and T2D (i.e. *SH2B3*, *MAPK14*, *CTRB1/2*, *INS*, *ASCL2/MIR4686*, and *HLA* region) using the COLOC R package. Briefly, T1D and T2D GWAS summary statistics of individuals of European ancestry were accessed from https://gwas.mrcieu.ac.uk/ using R software package (gwasglue; https://github.com/mrcieu/gwasglue/), and SNPs extracted within 200 kb from the lead SNP. Spatial *cis*-eQTL summary data was derived from CoDeS3D analysis as described in Sect. 5.2. For each SNP, we selected SNP-gene pairs with the lowest *p* value and performed colocalization (i.e. coloc.abf) test between disease and eQTL summary data with priors set as *p1* = 1 × 10^–4^, *p2* = 1 × 10^–4^, and *p12* = 5 × 10^–5^, as previously suggested^28^. In total, 2129 pairwise comparisons were examined for evidence of colocalization between eQTL and disease signals.

### Pathway analysis and functional gene annotations

Biological pathway enrichments for the differentially expressed genes were identified using the R software package (g:Profiler^27^) with a significance threshold of *p* value < 0.05 threshold. R software package (TissueEnrich^91^) was used for the tissue-specific gene expression analysis. PubTator Central^92^ was used for manual literature curation to examine the molecular and phenotypic implications of specific examples of differentially expressed genes.

### The construction of the PPI network for the pleiotropic genes

We used the Retrieval of Interacting Genes/Proteins database (STRING; v.11 9.0)^53^ to construct a protein–protein interaction network for the differentially expressed genes associated with both T1D and T2D. The PPI network was set to a medium confidence of 0.400 with the following prediction evidence: (1) genomic context prediction channels—neighborhood, fusion, and co-occurrence; (2) co-expression; (3) text mining; (4) curated databases; and (4) experiments. PPI clusters were identified using K-means clustering algorithm. The drug-gene interaction database (DGIdb; v3.0.2)^93^ was mined to identify genes that encode proteins that are targets for at least a single FDA-approved drug within the PPI network.

### Data analysis

Statistical testing, visualization, and genetic correlations analyses were performed using R software (v3.6.3) and RStudio (version 1.2.5042-1). Python version 3.7.6 was used for the bootstrap analysis. Scripts for genetic colocalization, data analysis and visualization can be accessed on Figshare with the identifier https://doi.org/10.17608/k6.auckland.12886745.

### Code and data accessibility

The CoDeS3D pipeline is available at: https://github.com/Genome3d/codes3d-v2/. HDL software is available at https://github.com/zhenin/HDL/. GWAS catalog can be accessed at http://www.ebi.ac.uk/gwas/. UKBB summary statistics from the Neale lab are available at https://www.nealelab.is/uk-biobank/. GTEx portal can be accessed at http://www.gtexportal.org/. The UCSC browser is accessed at https://genome.ucsc.edu/. The linkage disequilibrium matrix (LDlink) is available from https://ldlink.nci.nih.gov/. R software package, gwasglue, is accessed at https://github.com/MRCIEU/gwasglue. Circa software for generating circos plots is available at http://omgenomics.com/circa/. The STRING database can be accessed at http://string-db.org/. The Drug Gene Interaction database (DGIdb) can be accessed at http://www.dgidb.org/.

## Supplementary Information

Supplementary Information.

Supplementary Information 1.

## Data Availability

All data generated or analyzed during this study are included in this published article (and its Supplementary Information files [Supplementary Tables S1-S16]).
