# Supplementary material for: Untangling the genetic link between type 1 and type 2 diabetes using functional genomics
Source: Sci Rep. 2021 Jul 6;11:13871. doi: 10.1038/s41598-021-93346-x (PMC8260770; doi:10.1038/s41598-021-93346-x)
Supplement: Supplementary file 2 — Supplementary Information 1. [file 41598_2021_93346_MOESM2_ESM.docx]

**Supplementary Material**

Untangling the genetic link between type 1 and type 2 diabetes using functional genomics

**Denis M. Nyaga^1^, Mark H. Vickers^1^, Craig Jefferies^1,2^, Tayaza Fadason^1^, Justin M. O’Sullivan^1,3*^**

^1^Liggins Institute, The University of Auckland, Auckland, New Zealand

^2^Starship Children’s Health, Auckland, New Zealand

^3^The Maurice Wilkins Centre, The University of Auckland, Auckland, New Zealand

***Correspondence:**

Corresponding Author

[justin.osullivan@auckland.ac.nz](mailto:justin.osullivan@auckland.ac.nz)


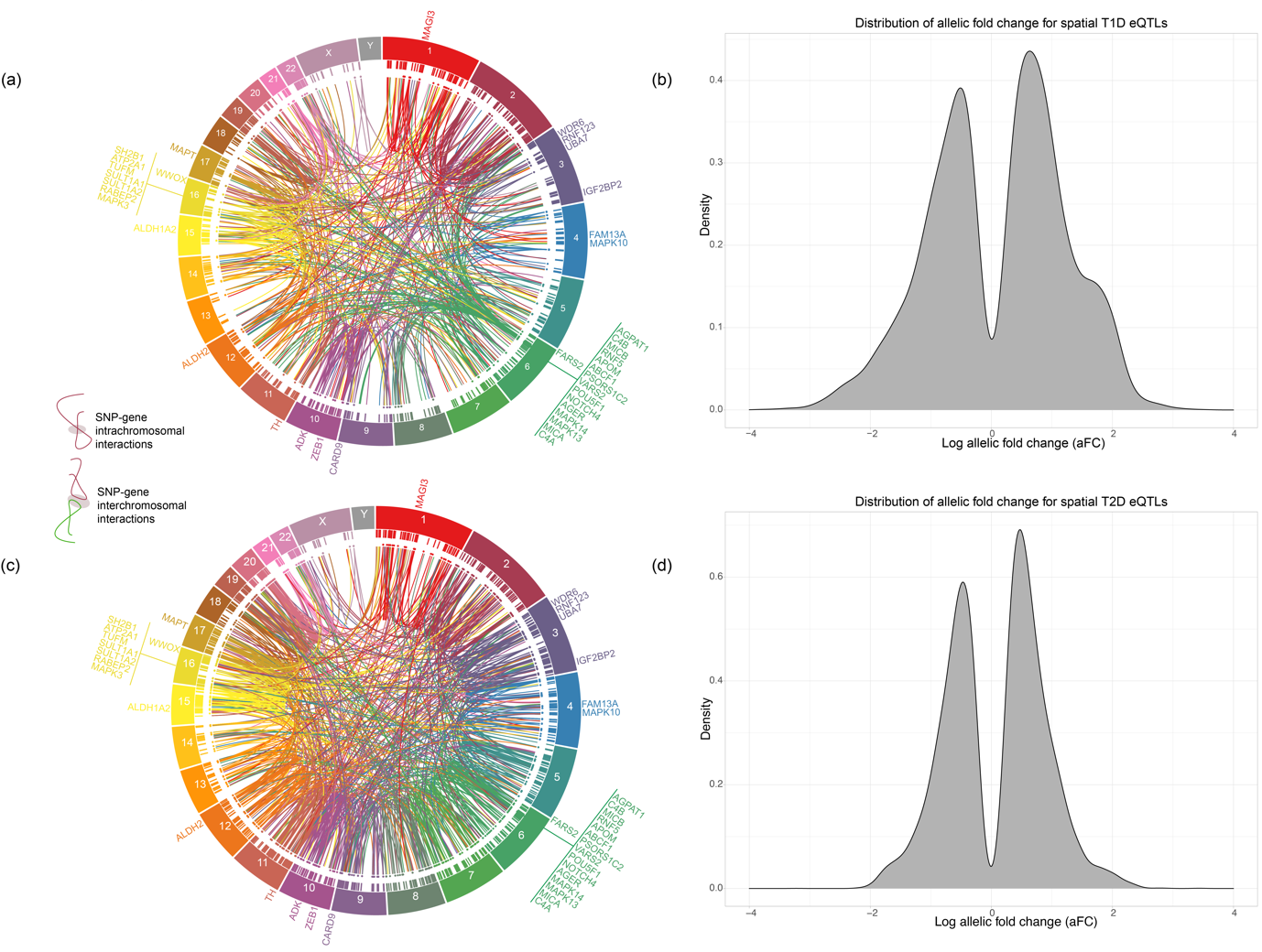


**Supplementary Figure S1.** **T1D and T2D-associated SNPs mark spatial gene regulatory loci which modulate gene expression levels**. **(a)** Circos plot showing spatial eQTL-eGene associations for T1D SNPs, and **(b)** the distribution of allelic fold change for the spatial T1D QTLs. **(c)** Circos plot showing spatial eQTL-eGene associations for T2D SNPs, and **(d)** the distribution of allelic fold change for the spatial T2D eQTLs. Data tracks for circos plots (from inner-most ring): link lines represent chromatin interactions (*i.e*. inter- and intrachromosomal interactions shown in the inset) between SNP fragments and the spatially regulated genes at FDR <0.05; scatter plot represents SNP positions, with the short lines representing the regulated genes. The outer most ring illustrate the somatic chromosomes, together with the x and y chromosomes, with the gene names (outside) showing examples of genes that mediate pleiotropic effects on both T1D and T2D. eQTL; expression quantitative trait loci, eGene; a gene whose transcript levels is associated with an eQTL. Circa software was used to generate the circos plot and is available at http://omgenomics.com/circa/.


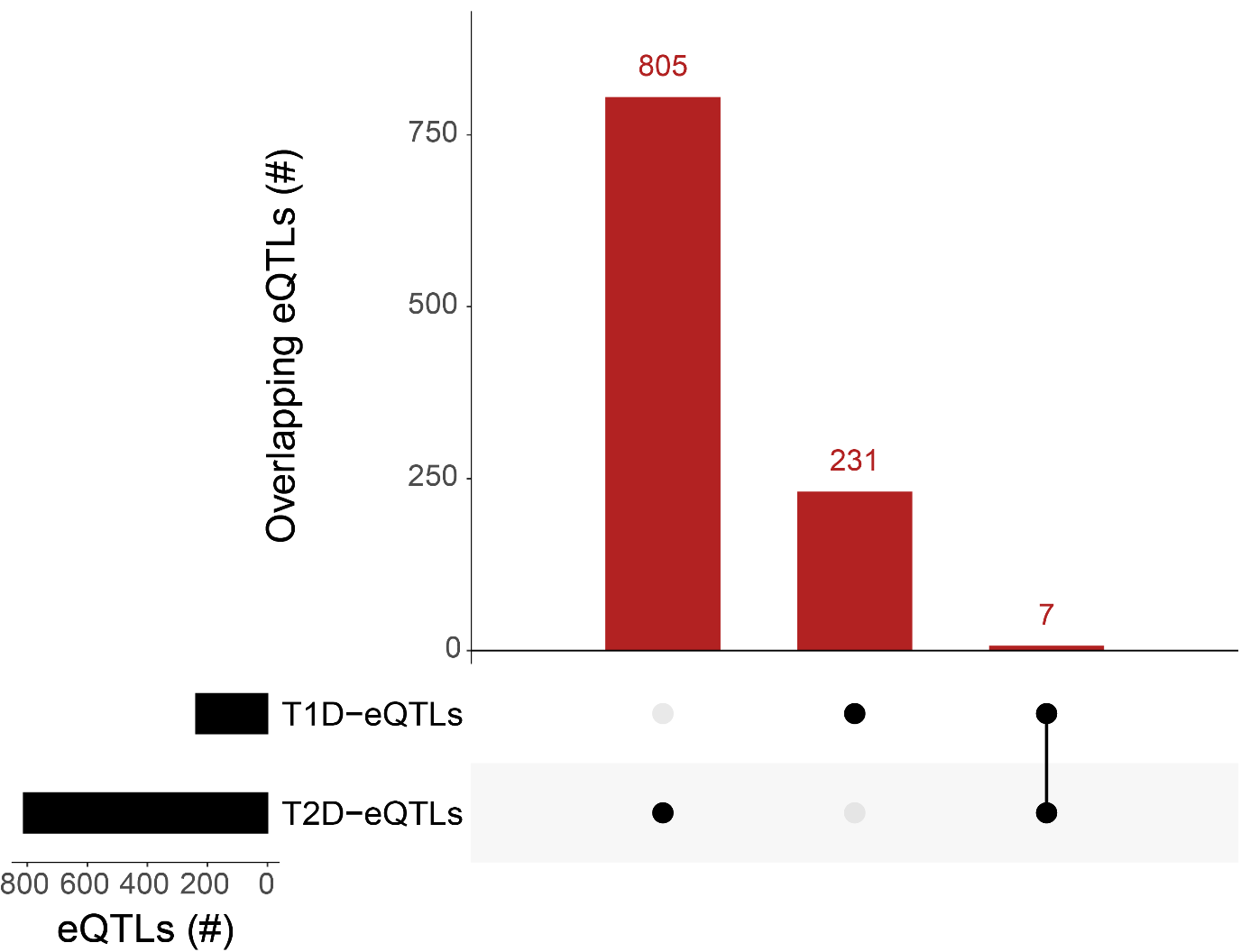


**Supplementary Figure S2.** Only 7 of the 12 SNPs that were associated with both T1D and T2D mark spatial regulatory loci as identified by CoDeS3D. 805 eQTLs are unique for T2D, while 231 eQTLs are unique for T1D.


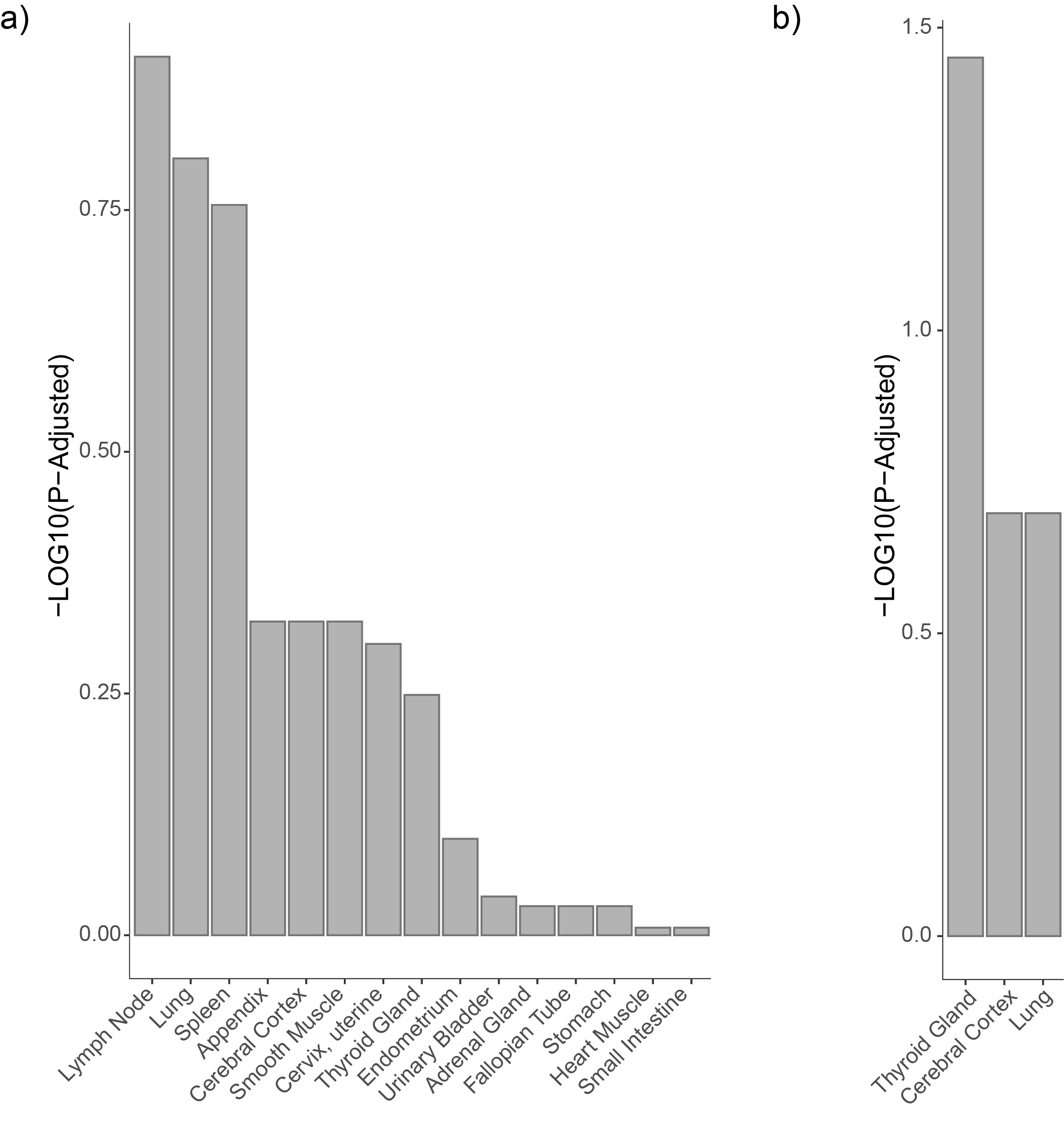


**Supplementary Figure S3. Tissue-specific enrichment eGenes**. Enrichment of **(a)** T1D and **(b)** T2D eGenes using R package – TissueEnrich [1]. Thyroid tissue was identified as having the highest level of enrichment for expression of the genes that were regulated by eQTLs associated with T2D, while lymph nodes, lung and spleen were the most enriched tissues for T1D. The human protein atlas (HPA) RNA-seq dataset was set as the reference panel for tissue enrichment.

**References**

[1] A. Jain, G. Tuteja, TissueEnrich: Tissue-specific gene enrichment analysis, Bioinformatics. 35 (2019) 1966–1967. https://doi.org/10.1093/bioinformatics/bty890.
